# Supplementary material for: Adult-Onset CNS Sulfatide Deficiency Causes Sex-Dependent Metabolic Disruption in Aging
Source: Int J Mol Sci. 2023 Jun 22;24(13):10483. doi: 10.3390/ijms241310483 (PMC10341976; doi:10.3390/ijms241310483)
Supplement: Supplementary file 1 [file ijms-24-10483-s001.zip › ijms-2448307-supplementary.pdf]

Supplementary Figures

Adult-onset CNS sulfatide deficiency causes sex-dependent metabolic disruption in aging

Shulan Qiu[1,2,†], Sijia He[1,†], Jianing Wang[1,‡], Hu Wang[1], Anindita Bhattacharjee[1], Xin Li[1], Moawiz Saeed[1], Jeffrey L. Dupree[3,4], and Xianlin Han [1,2,*]

[1]Barshop Institute for Longevity and Aging Studies, University of Texas Health Science Center at San Antonio, San Antonio, TX 78229, USA

[2]Division of Diabetes, Department of Medicine, University of Texas Health Science Center at San Antonio, San Antonio, TX 78229, USA

[3]Department of Anatomy and Neurobiology, Virginia Commonwealth University, Richmond, VA 23284, USA

[4]Research Division, McGuire Veterans Affairs Medical Center, Richmond, VA 23249, USA

* Correspondence: hanx@uthscsa.edu

† These authors have equal contribution to this study.

‡ Present address of Jianing Wang: State Key Lab. of Environmental & Biological Analysis, Department of Chemistry, Hong Kong Baptist University, Hong Kong, China.

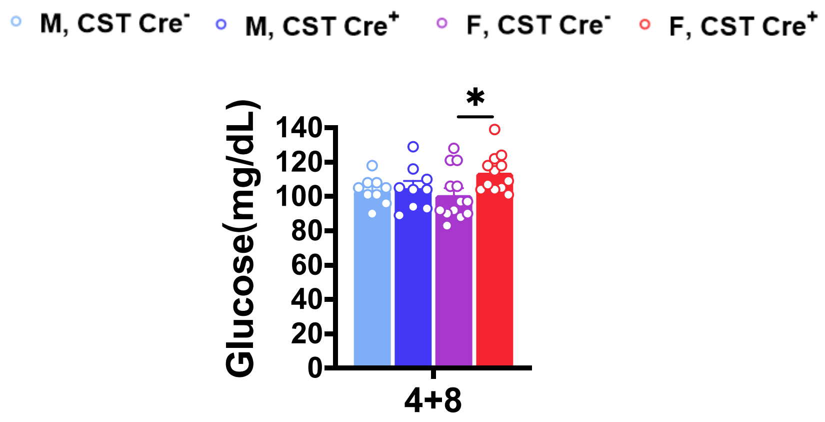

Figure S1. Blood glucose levels measured without fasting at 8 months post tamoxefin injection under normal diet. n=9-14. Multiple t-Test.

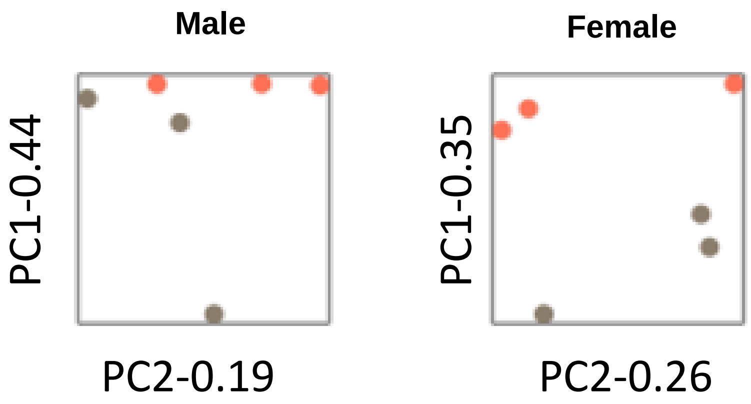

Figure S2. PCA showing that female CST Cre+ cluster in a more distinct manner versus Cre-female mice relative to male CST Cre+ and Cre- mice

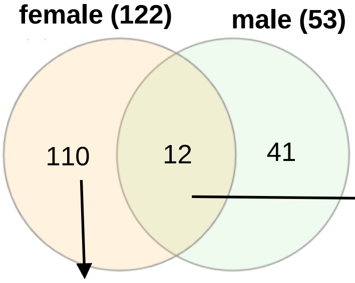

Bcl2a1a	*Ccl3*	*Ccl4*	*Cd86*
Ch25h	*Gal3st1*	*lag3*	*Mmp12*
Opalin	*Ptpn6*	*Serina3n*	*Timp1*

Apoe	*Atf3*	*Axl*	*Bag4*	*Bcl10*	*Blnk*	*Bok*	*C1qa*	*C1qb*	*C1qc*
C3	*C3ar1*	*C4a*	*C5ar1*	*Cables1*	*Casp7*	*Ccl2*	*Ccl5*	*Ccl7*	*Cd109*
C4	*Cd33*	*Cd68*	*Cd72*	*Cd84*	*Clec7a*	*Cotl1*	*Csf1*	*Csf1r*	*Csf3r*
Cst7	*Ctss*	*Cx3cr1*	*Dock2*	*Erbb3*	*F3*	*Fabp5*	*Fbln5*	*Fcer1g*	*Fcgr1*
Fcgr2b	*Fcgr3*	*Fcrls*	*Fgd2*	*Gadd45a*	*Gadd45g*	*Gpr183*	*Gpr34*	*Grn*	*Hmox1*
Hsd11b1	*Hspb1*	*Icam2*	*Ifi30*	*Igsf6*	*Il2rg*	*Il3ra*	*Inpp5d*	*Irak2*	*Irf8*
Itgax	*Itgb5*	*Jag1*	*Lilrb4a*	*Ly9*	*Lyn*	*Mafb*	*Man2b1*	*Mbd3*	*Mcm2*
Mef2c	*Mpeg1*	*Mr1*	*Msn*	*Mvp*	*Myc*	*Ncf1*	*Nfkb2*	*Osmr*	*Pdpn*
Pla2g4a	*Plcg2*	*Pros1*	*Psmb8*	*Rad51c*	*Relb*	*Rsad2*	*S100a10*	*S1pr3*	*Siglecf*
Slamf9	*Slc10a6*	*Slco2b1*	*Sox9*	*Spp1*	*Tcirg1*	*Tgfb1*	*Tgfbr1*	*Tgm1*	*Tlr2*
Tlr7	*Tm4sf1*	*Tmem204*	*Tnfrsf1a*	*Traf2*	*Trem2*	*Trp53*	*Tyrobp*	*Vav1*	*Vim*

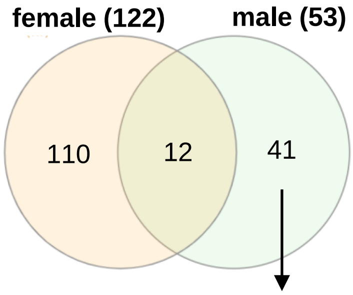

Atg3	Atg5	Atm	Brd2	Casp8	Cd69	Cd70	Creb1	Csk	Ctse
E2f1	Esam	Fcrla	Fdxr	Foxp3	Gpr84	Igf1	Il21r	Irf2	Irf7
Islr2	Itga6	Kcnk13	Kdm4a	Klrk1	Kmt2c	Lig1	Ly6a	Myct1	Pnoc
Ptx3	Rab6b	Ripk1	Setd7	Sin3a	Slamf8	Snca	Tmem119	Tnfrsf11b	Tnfsf8
Vps4a									

Figure S3. DEGs lists in Venn diagrams from female or male CST Cre^{+} vs. CST Cre^{-}.
